# Glymphatic system in neurological disorders and implications for brain health

**DOI:** 10.3389/fneur.2025.1543725

**Published:** 2025-02-05

**Authors:** Osman Corbali, Allan I. Levey

**Affiliations:** Department of Neurology, Emory University School of Medicine, Atlanta, GA, United States

**Keywords:** glymphatic system, perivascular space, slow wave sleep, brain health, dementia, CSVD, stroke

## Introduction

Nearly a decade ago, a paravascular pathway, enabling cerebrospinal fluid (CSF) to flow through the brain parenchyma, was discovered (1). Known as the “glymphatic” (glial-lymphatic) system, it has emerged as a critical process for clearing waste from the brain's interstitial tissue, which lacks histologically distinct lymphatic vessels (2).

**Figure 1:**
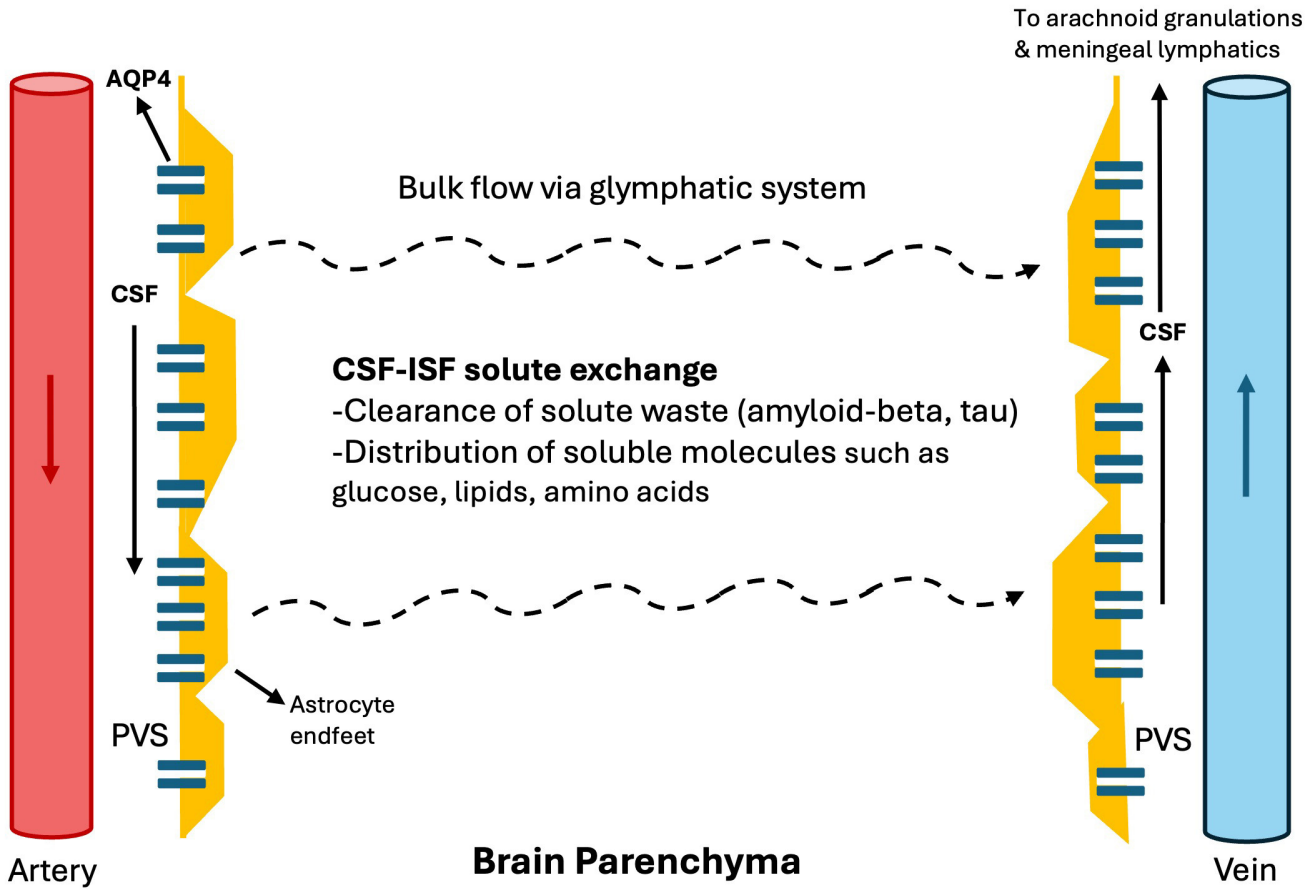
Glymphatic system. In the glymphatic system, arterial pulsations propagate CSF through the perivascular spaces as penetrating arteries arise from major arteries within the subarachnoid cisterns. These PVS are formed by the vascular endfeet of astrocytes, which facilitate CSF flow through the abundant expression of AQP4. The CSF then diffuses through the brain's interstitial fluid and exits via perivenous spaces, draining either through meningeal and cervical lymphatic vessels or arachnoid granulations. CSF: cerebrospinal fluid, PVS: perivascular space. ISF, Interstitial fluid.

In addition to the conventional CSF circulation from the choroid plexus through arachnoid granulations and venous sinuses; in the glymphatic system (Figure 1), arterial pulsations propagate CSF through the perivascular spaces (PVS) (2). These PVS are formed by the vascular endfeet of astrocytes, which facilitate CSF flow through the abundant expression of aquaporin-4 (AQP4) (1–3). The CSF then diffuses through the brain's interstitial fluid and exits via perivenous spaces, draining either through meningeal and cervical lymphatic vessels or arachnoid granulations (4, 5).

The glymphatic system was initially discovered in rodent models (1), and its presence has also been demonstrated in humans (6–8) through intrathecal contrast MRI studies by demonstrating contrast flow through the subarachnoid spaces, perivascular spaces, and finally into the brain parenchyma (7, 8).

Interestingly, the glymphatic system is most active during non-rapid eye movement (NREM) sleep, particularly N3 stage (9, 10). Human studies support that glymphatic activity is enhanced during sleep, increasing with higher delta activity and decreasing with higher beta activity and heart rate (11). Moreover, sleep position influences glymphatic efficiency (lateral more efficient than supine or prone) (12).

The glymphatic system plays a vital role in clearing soluble proteins and brain metabolites, facilitating fluid and solute exchange across the brain parenchyma. This includes glucose for energy metabolism, lipid transport, signaling molecules, and the removal of waste products such as amyloid-beta and tau, as demonstrated in both animal models and humans (1, 5, 13–17). In humans, sleep deprivation impairs CSF-to-blood clearance of amyloid-beta and tau (17), and a sleep-active glymphatic system contributes to the clearance of these proteins from the brain (14). Demonstration of the glymphatic system in humans is a breakthrough that connects aging, sleep, cardiovascular health, and brain health.

## Measurement of glymphatic function in humans

Multiple approaches have been proposed to measure glymphatic activity or dysfunction in humans (18). The glymphatic system was initially demonstrated through intrathecal contrast-based studies, which revealed a centripetal enhancement pattern moving from the subarachnoid space into the parenchyma (gray and white matter) along the perivascular spaces (7, 8, 19).

Subsequently, intravenous contrast-based methods were also developed. These techniques employ region-of-interest analyses of the brain parenchyma and account for both vascular and CSF contributions to parenchymal enhancement (11, 20). However, IV or intrathecal contrast methods often require sequential MRI measurements to track contrast flow.

Another widely used approach is diffusion-derived MRI, specifically the assessment of diffusivity along the perivascular space (ALPS) (21, 22). This method is quick, non-invasive, does not require contrast; however, it does not capture the entire glymphatic system (i.e., gray matter). Instead, it relies on diffusion tensor imaging of white matter at the level of the lateral ventricles (23).

Enlarged perivascular spaces (ePVS) have been proposed as an indirect marker of glymphatic dysfunction and can be visualized on conventional MRI, particularly T2-weighted sequences (18). Still, they may reflect diverse pathophysiological processes rather than glymphatic function specifically. For instance, basal ganglia ePVS are commonly associated with lacunar strokes, whereas centrum semiovale ePVS often correlate with cerebral amyloid angiopathy (24, 25).

Various other methods to evaluate glymphatic function are discussed elsewhere (18). A key challenge in the field is the development of techniques that are minimally invasive, widely accessible, and capable of real-time assessment.

More recently, an investigational non-invasive device for measuring brain parenchymal resistance (Rp) was introduced, offering a promising method for predicting sleep-active glymphatic function (11). This approach demonstrated that glymphatic activity in humans also increases with higher EEG delta power, lower EEG beta power, and reduced heart rate—mirroring findings in animal models (9). Because this device relies on measuring Rp, it provides high temporal resolution (nearly continuous measurement) but has limited anatomical information.

## Dysfunction of glymphatic system in neurological diseases

### Dementia and cognitive impairment

The glymphatic system plays a crucial role in clearing amyloid-beta and tau from CSF and the brain (14, 16, 17). Consequently, dysfunction of glymphatic system can contribute to the pathophysiology of dementia (2). Furthermore, glymphatic dysfunction and dementia share common risk factors, including aging, sleep abnormalities, and cardiovascular diseases. For example, in older adults or individuals with dementia, NREM sleep—the stage when the glymphatic system is most active—tends to decrease (26, 27). In addition, obstructive sleep apnea, a modifiable risk factor for Alzheimer's disease (AD) and Parkinson's disease (PD) (28), has been associated with reduced glymphatic function (demonstrated by a low ALPS index) (29, 30).

Glymphatic dysfunction has been observed in both AD and PD. Studies indicate reduced ALPS indices in individuals with AD, including those in prodromal and preclinical stages, and these lower ALPS values predict an accelerated accumulation of amyloid-beta on PET imaging (31–33). In PD, a decreased ALPS index is associated with more rapid clinical deterioration, as measured by MDS-UPDRS parts II and III, as well as the Symbol Digit Modalities Test (34, 35). Moreover, a recent meta-analysis encompassing 11 studies on AD and 12 studies on PD provided strong evidence for reduced ALPS indices in both diseases (36).

Additionally, Eide et al. (37) demonstrated impaired glymphatic function in normal pressure hydrocephalus (NPH) through an intrathecal contrast-based MRI study involving 30 patients with idiopathic NPH and 8 control patients. Their findings revealed delayed CSF contrast clearance in NPH, supporting the notion that diminished amyloid-beta clearance may contribute to cognitive dysfunction in this population.

### Stroke

Stroke risk factors, such as diabetes and hypertension, are associated with glymphatic dysfunction (38–41), as discussed in the next section. In turn, stroke itself can cause ipsilateral glymphatic impairment (42). Glymphatic dysfunction could also be a stroke risk factor; because ePVS is associated with increased stroke risk (5, 43); however, this relationship warrants further investigation, given that ePVS is only an indirect marker of glymphatic function.

Glymphatic impairment following stroke predominantly occurs on the side of the infarct (42, 44). Toh et al. (42) reported decreased ALPS indices ipsilateral to the stroke location in patients with ischemic stroke (*n* = 50) compared with controls (*n* = 44), and ALPS index inversely correlated with stroke size and improved over time. In a smaller cohort (*n* = 18) with large-vessel occlusion (LVO), Zhu et al. (44) corroborated these findings, demonstrating impaired ipsilateral glymphatic function early after stroke (days 1 and 3), which then recovered by day 7.

Zhu et al. (44) also proposed that glymphatic function could modulate the extent of brain edema following ischemic stroke. Using a rodent middle cerebral artery occlusion (MCAO) model, they found that glymphatic recovery coincided with improvements in post-stroke edema, despite a worsening of blood–brain barrier dysfunction by day 7 (44, 45). Moreover, pharmacological enhancement of glymphatic activity via adrenergic receptor antagonists alleviated edema by day 2, reduced amyloid-beta deposition, and improved cognitive function (44).

Furthermore, glymphatic dysfunction may play a role in post-stroke epileptogenesis and cognitive impairment (46), potentially through glutamate excitotoxicity and amyloid-beta/tau deposition. EPVS asymmetry has been linked to focal seizures (47), but longitudinal studies in stroke patients are needed to establish the relationship between asymmetric ePVS and the occurrence of focal seizures.

Glymphatic dysfunction is also evident in hemorrhagic stroke—indicated by an increased ePVS burden—and in subarachnoid hemorrhage, where preclinical models have shown altered AQP4 polarization (5). These conditions are discussed in more detail elsewhere (5).

### Cerebral small vessel disease

Cerebral small vessel disease (CSVD) and glymphatic dysfunction share common risk factors and etiologies, including hypertension, diabetes, aging, sleep disruption, and neuroinflammation (48). EPVS acts as a marker of CSVD (49) and also serve as an indirect marker of glymphatic dysfunction (18). In older adults, ePVS is associated with the progression of white matter hyperintensities, subcortical infarcts, microbleeds, and vascular dementia (49, 50).

CSVD is generally categorized into two pathophysiological subtypes: amyloid [cerebral amyloid angiopathy (CAA)] and non-amyloid (hypertensive arteriopathy), both of which can present with microbleeds and microinfarcts (51). In the amyloid subtype, CAA is associated with a lower ALPS index, and reduced glymphatic function correlates with a higher CSVD burden, poorer cognitive performance, larger white matter hyperintensities, white matter lacunes, and disease recurrence (52). In non-amyloid CSVD, in addition to lesions (e.g., microinfarcts) that may contribute to glymphatic impairment (53), arteriosclerosis may theoretically further impair the glymphatic system by affecting arterial pulsatility (54).

Recent evidence also indicates that glymphatic dysfunction is linked to cognitive impairment in CSVD (55). Among 133 patients with CSVD, those with cognitive impairment (*n* = 83) showed a lower ALPS index compared to cognitively normal CSVD individuals (*n* = 50), after adjusting for multiple variables including other CSVD imaging markers. The ALPS index was positively associated with cognitive test scores (e.g., MoCA, AVLT-sum, SDMT) and negatively correlated with TMT B–A scores (55).

### Multiple sclerosis

Glymphatic dysfunction has been observed in MS patients (21, 56). Cartenuto et al. (21) reported lower ALPS index in MS patients compared to HC, and progressive MS with even further lower ALPS compared to relapsing-remitting MS. Potential mechanisms for glymphatic disruption in MS include altered CSF dynamics, abnormal AQP4 expression near lesion sites, a predilection for lesions in perivenular areas, and impaired meningeal lymphatic vessels affecting CSF outflow (57).

### Traumatic brain injury

A lower ALPS index has been reported in patients with traumatic brain injury, and potential mechanisms include sleep impairment and loss of polarized location of AQP4 (58). In patients with TBI, the ALPS index negatively correlating with serum neurofilament levels (59). This suggests that greater axonal injury is associated with more severe glymphatic dysfunction.

### Migraine

Preliminary studies have shown no significant decrease in the DTI-ALPS index among migraine patients (60, 61). However, two recent studies report varying glymphatic function in chronic migraine (62, 63). Further research is needed to clarify the role of the glymphatic system in migraine pathophysiology; it may act as an important modifier by serving as a sink for calcitonin gene-related peptide (64).

### Idiopathic intracranial hypertension

Lower glymphatic function has been demonstrated in patients with idiopathic intracranial hypertension (IIH) using intrathecal contrast MRI (15 IIH patients and 15 controls) (65). The study revealed delayed parenchymal clearance of the CSF tracer, particularly in regions such as the hippocampus and entorhinal cortex, which are susceptible to amyloid-beta and tau deposition and are associated with Alzheimer's disease (AD).

## Controlling comorbidities to improve glymphatic system

### Sleep disorders

Poor sleep quality is a known trigger for migraines, provoked seizures, and delirium, and it is commonly observed in neurodegenerative disorders and depression. The glymphatic system may serve as a critical link between sleep and brain health.

During wakefulness, higher levels of norepinephrine increase brain parenchymal resistance, thereby suppressing glymphatic activity (10, 66). Interestingly, locally blocking norepinephrine receptors in awake mice significantly enhances glymphatic function, bringing it closer to the levels observed during sleep or anesthesia (10, 66).

Sleep architecture is an important determinant of glymphatic function, with the system being most active during NREM sleep, particularly in stage N3 when delta activity is highest. Disruption of N3 sleep has been associated with conditions such as dementia (26, 67), highlighting the potential benefit of interventions aimed at improving slow-wave sleep (67).

Addressing insomnia is thus essential for brain health, and future pharmacological and non-pharmacological interventions should be evaluated for their effects on sleep architecture and, in turn, glymphatic function. For example, in a randomized trial comparing melatonin, temazepam (a benzodiazepine), zolpidem (a benzodiazepine-like drug), and placebo, temazepam and zolpidem significantly decreased NREM slow-wave activity on EEG compared to placebo, whereas melatonin did not significantly alter overall slow-wave activity (68).

Other studies also indicate that benzodiazepines drugs disrupt sleep architecture by reducing delta activity and N3 sleep, which may explain why these agents, despite increasing total sleep duration, fail to improve daytime functioning (26, 69–71). Furthermore, benzodiazepines have been associated with brain atrophy in regions such as the hippocampus and amygdala (72).

Non-pharmacological interventions, such as acoustic stimulation, transcranial direct current stimulation, and transcranial magnetic stimulation, are also demonstrated to enhance slow-wave sleep (26, 73–75). Future research into these non-invasive methods and their impact on glymphatic function will be particularly valuable.

Finally, sleep-related breathing disorders can also play a relevant role in glymphatic dysfunction. For example, in patients with obstructive sleep apnea, the ALPS index has been reported to decrease, correlating with disease severity (29, 30).

### Depression

Depression is frequently accompanied by insomnia and linked to increased REM sleep and decreased slow-wave sleep (76). The effects of antidepressant medications on the glymphatic system are not well studied. Nevertheless, antidepressants have varying impacts on sleep architecture. For example, SSRIs and SNRIs typically increase REM latency, may have mixed effects on slow-wave sleep, and can reduce sleep continuity in the short term (77). Clinicians should be mindful of these effects when managing sleep disturbances in patients with depression.

### Cardiovascular diseases

Cardiovascular health is critical for glymphatic function, as it relies on arterial pulsatility to drive CSF through PVS and into the interstitial fluid (ISF) of the brain.

Cardiovascular diseases that impair cardiac output, such as heart failure with reduced ejection fraction or arrhythmias, as well as conditions that reduce arterial wall elasticity—such as arteriosclerosis, hypertension, small vessel disease, and atherosclerosis—can theoretically disrupt CSF flow through the PVS in the glymphatic (2). In humans, hypertension is associated with glymphatic dysfunction, reflected by a lower ALPS index (41). Similarly, in mouse models, hypertension impairs glymphatic function, increases ePVS, and disrupts AQP4 polarity (54).

Diabetes, another significant cardiovascular risk factor, has also been associated with a decreased ALPS index (38–40). Moreover, insulin resistance has been shown to negatively correlate with glymphatic function as well (39).

### Importance of exercise

Exercise improves overall cardiovascular health and can enhance glymphatic system during both the awake and sleeping states (78–81), through mechanisms such as improved cardiovascular dynamics and sleep quality.

Regular exercise lowers resting heart rate (82), which is associated with higher glymphatic function (11). Exercise also helps regulate hypertension, a condition linked to glymphatic dysfunction, though the causal relationship remains unclear (41). Preclinical mouse studies further support that voluntary running improves glymphatic function, except during active exercise when the brain deprioritizes waste clearance, likely due to the inhibitory effects of norepinephrine (78, 80).

Exercise also impacts sleep architecture. A recent study found that both low-intensity and moderate-to-vigorous physical activity increased NREM sleep in humans, decreased REM sleep, and extended REM latency (81).

## Discussion

**Figure 2:**
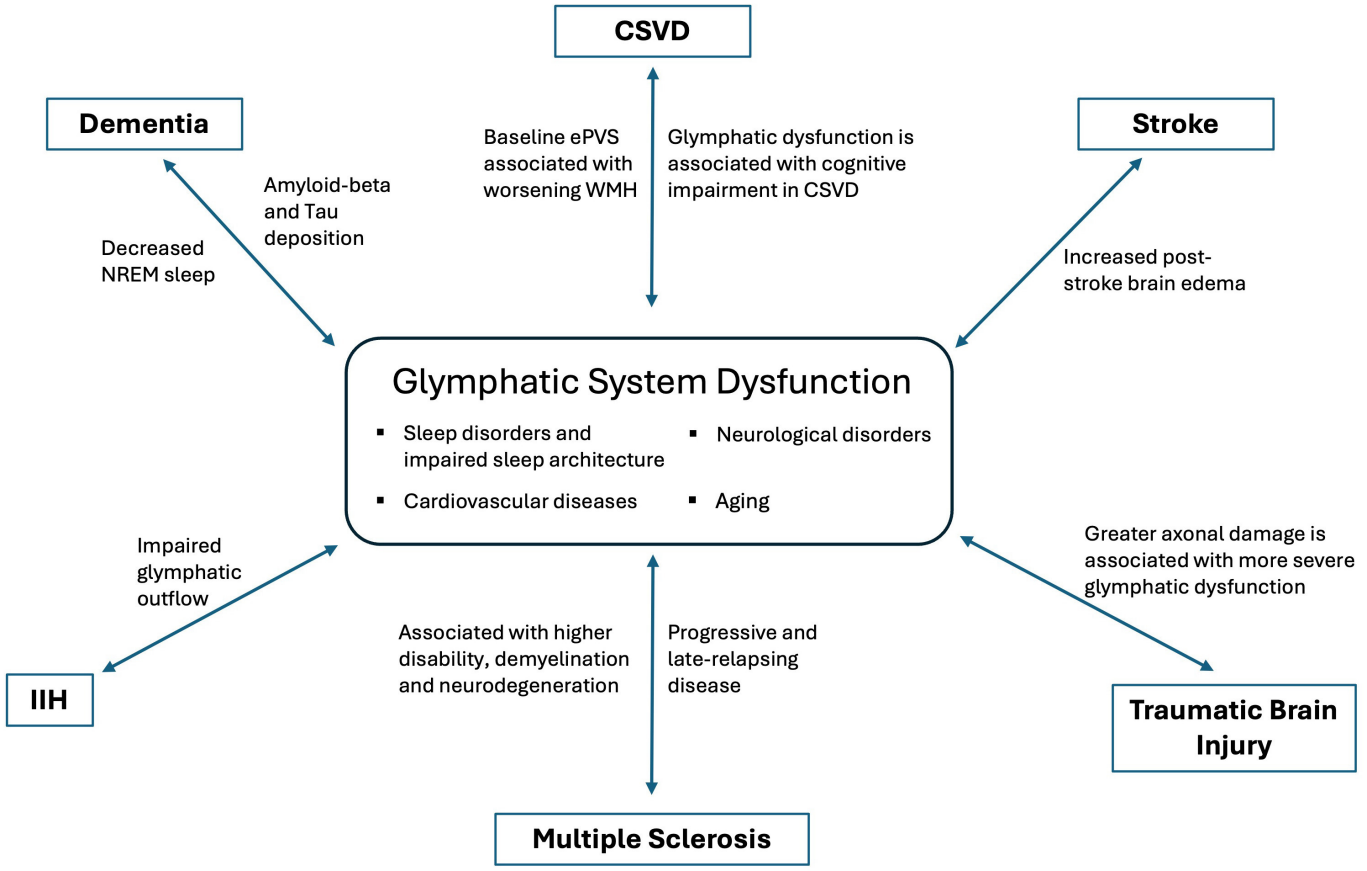
Dysfunction of glymphatic system in various neurological disorders.

The glymphatic system offers a novel framework for understanding the interplay between sleep, exercise, cardiovascular health, and brain function. Glymphatic dysfunction has been implicated in numerous neurological diseases (Figure 2), highlighting the need for further research to determine whether augmenting glymphatic activity can prevent or improve disease outcomes. From neuromodulation in post-stroke recovery to reducing toxic solute burden in neurodegenerative diseases and modulating the neuro-inflammatory milieu, the glymphatic system represents a promising area for clinical neurology.

Advancing this field requires two key steps. First, the development of faster, more accessible, and non-invasive tools to assess glymphatic function in humans is crucial, and progress is already underway (11). Second, more studies are needed to directly target glymphatic function—whether through revisiting established techniques like acoustic stimulation or exploring pharmacological and biological approaches, such as enhancing AQP4 functionality (83).
